# Phosphorylation of GAP-43 T172 is a molecular marker of growing axons in a wide range of mammals including primates

**DOI:** 10.1186/s13041-021-00755-0

**Published:** 2021-04-08

**Authors:** Masayasu Okada, Yosuke Kawagoe, Yuta Sato, Motohiro Nozumi, Yuya Ishikawa, Atsushi Tamada, Hiroyuki Yamazaki, Yuko Sekino, Yonehiro Kanemura, Yohei Shinmyo, Hiroshi Kawasaki, Naoko Kaneko, Kazunobu Sawamoto, Yukihiko Fujii, Michihiro Igarashi

**Affiliations:** 1 Department of Neurosurgery, Brain Research Institute, School of Medicine and Graduate School of Medical/Dental Sciences, Niigata University, Niigata, Japan; 2 Medical and Dental Hospital, School of Medicine and Graduate School of Medical/Dental Sciences, Niigata University, Niigata, Japan; 3 Departments of Neurochemistry and Molecular Cell Biology, School of Medicine and Graduate School of Medical/Dental Sciences, Niigata University, Niigata, 951-8510 Japan; 4 Department of Orthopedic Surgery, School of Medicine and Graduate School of Medical/Dental Sciences, Niigata University, Niigata, Japan; 5 Department of Neurobiology and Behavior, Gunma University Graduate School of Medicine, Maebashi, Japan; 6 Laboratory of Chemical Pharmacology, Graduate School of Pharmaceutical Sciences, University of Tokyo, Tokyo, Japan; 7 Division of Regenerative Medicine, Department of Biomedical Research and Innovation, Institute for Clinical Research, National Hospital Organization Osaka National Hospital, Osaka, Japan; 8 Department of Medical Neuroscience, Graduate School of Medical Sciences, Kanazawa University, Kanazawa, Japan; 9 Department of Developmental and Regenerative Neurobiology, Institute of Brain Science, Nagoya City University Graduate School of Medical Sciences, Nagoya, Japan; 10 Division of Neural Development and Regeneration, National Institute for Physiological Sciences, Okazaki, Japan; 11 Present Address: Department of Chemistry, Faculty of Science, Hokkaido University, Sapporo, 060-0810 Japan; 12 Present Address: Department of iPS Cell Applied Medicine, Faculty of Medicine, Kansai Medical University, Hirakata, Osaka 573-1010 Japan

**Keywords:** Phosphorylation, GAP-43, JNK, Brain development, Axon growth, Axon regeneration, Primates

## Abstract

GAP-43 is a vertebrate neuron-specific protein and that is strongly related to axon growth and regeneration; thus, this protein has been utilized as a classical molecular marker of these events and growth cones. Although GAP-43 was biochemically characterized more than a quarter century ago, how this protein is related to these events is still not clear. Recently, we identified many phosphorylation sites in the growth cone membrane proteins of rodent brains. Two phosphorylation sites of GAP-43, S96 and T172, were found within the top 10 hit sites among all proteins. S96 has already been characterized (Kawasaki et al., 2018), and here, phosphorylation of T172 was characterized. In vitro (cultured neurons) and in vivo, an antibody specific to phosphorylated T172 (pT172 antibody) specifically recognized cultured growth cones and growing axons in developing mouse neurons, respectively. Immunoblotting showed that pT172 antigens were more rapidly downregulated throughout development than those of pS96 antibody. From the primary structure, this phosphorylation site was predicted to be conserved in a wide range of animals including primates. In the developing marmoset brainstem and in differentiated neurons derived from human induced pluripotent stem cells, immunoreactivity with pT172 antibody revealed patterns similar to those in mice. pT172 antibody also labeled regenerating axons following sciatic nerve injury. Taken together, the T172 residue is widely conserved in a wide range of mammals including primates, and pT172 is a new candidate molecular marker for growing axons.

## Introduction

Growth-associated protein of 43-kDa (GAP-43, also known as neuromodulin) is a neuron-specific protein in vertebrates and is related to axon growth and regeneration [1–4]. Thus, this protein is considered a classical molecular marker of the growth cone in cultured neurons and regenerating axons in vivo [1, 4]. In the adult brain, GAP-43 is mainly localized in the presynaptic terminals and may be involved in neuronal plasticity [2].

Biochemically, GAP-43 has an IQ motif that binds to calmodulin in a Ca^2+^-independent manner [5]. The N*-*terminus of this protein is palmitoylated at C3 and C4, and its first 10 amino acids corresponding to the first coding exon are thought to be necessary and sufficient for its attachment to the membrane [6]. In particular, overexpression of this protein induces filopodia in vitro [7, 8] and sprouting in vivo [9], suggesting that GAP-43 is closely related to axon growth in the developing vertebrate brain. Although such a phenomenon related to GAP-43 suggests that this protein is involved in important signaling pathways during axon growth or regeneration, this is not clearly understood.

We have recently applied phosphoproteomics, a powerful technique to identify all phosphorylation sites of the proteome in a given system [10], to growth cone membranes derived from the rodent brain, and identified more than 1200 phospho-sites, most of which are not fully characterized [11–13]. Among these, phosphorylation of GAP-43 at S96, the top hit site, has been characterized and is tightly associated with axon growth and axon regeneration [11–14]. We identified another phosphorylation site of GAP-43, T172, in rodents. T172 is the second most abundant GAP-43 phosphorylation site and the ninth highest in the entire phosphoproteome [12, 13]. Here, we characterized this site using phospho-specific antibodies.

In vitro (cultured cortical neurons) and in vivo, an antibody specific to phosphorylated T172 (pT172Ab) specifically recognized cultured growth cones and growing axons, respectively, in developing mouse brain neurons. The pT172Ab antigens were more rapidly downregulated throughout development than the pS96Ab antigens, as seen with immunoblotting. Because this phosphorylation site was predicted to be conserved in a wide range of animals including primates, we performed immunohistochemistry and immunofluorescence studies in primates. In the developing marmoset brainstem and in differentiated neurons derived from human induced pluripotent stem cells (hiPSCs), immunoreactivity with pT172Ab revealed patterns similar to those in mice. Taken together, the T172 residue is widely conserved in a wide range of mammals including primates, and phosphorylated T172 is a new candidate molecular marker for growing axons.

## Results

### pT172 of GAP-43 is detected in growth cones of cultured mouse neurons

**Fig. 1:**
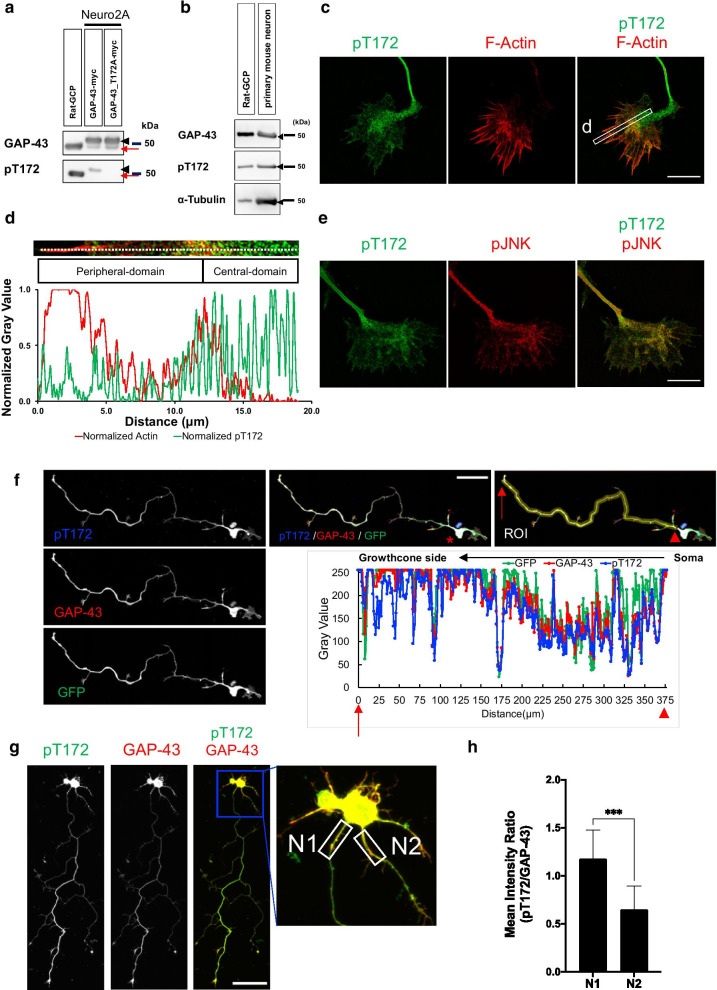
pT172Ab specifically reacted with phosphorylated T172 of GAP-43. **a** pT172Ab recognized the phosphorylated form of GAP-43 in the lysate of Neuro 2A cells that were transfected with wild-type GAP-43 construct, but the Ab did not recognize mutant T172A GAP-43. *GCP*: rat growth cone particle lysate (0.8 μg). *Arrowhead*: overexpressed GAP-43; *arrow (red):* endogenous GAP-43 of Neruo 2A cells. **b** Immunoblotting showed that pT172Ab reacted with rat GCP [12, 13] and the lysate of E15 cultured mouse cortical neurons (3 days in vitro (DIV3)). *Arrowhead*s: GAP-43, pT172, and α-tubulin immunoreactivity. **c** SIM super-resolution imaging of pT172Ab staining of phospho-GAP-43 T172 showed enriched expression in the C-domain compared to the P-domain. The white box indicates the ROI that was measured in (**d**). **d** Quantitative distribution of the ROI in (**c**) by measuring the fluorescence intensity along the white dashed line from the filopodial tip. Each fluorescence intensity of F-actin and of pT172 was measured by gray value, and the maximum value of each fluorescence was set to 1.0 for standardized and normalized measurement. **e** Colocalization of pT172 and phospho-JNK (pJNK) observed using SIM super-resolution microscopy. They were mainly located in the C-domain and partially in filopodia. Scale bar: 10 μm. **f** Z-stack maximum-intensity projection images of the mouse hippocampal neurons. Immunofluorescence using Abs of pT172 and GAP-43. *Upper right*: ROI of the gray values from the soma to the growth cone of (**f**) (*lower graph*). *Lower graph*: quantitative distribution of the ROI along the cell body to the growth cone. *Arrowheads*: growth cone. *Arrows*: neuronal soma. **g** Immunofluorescent studies of cultured mouse hippocampal neurons (DIV3) using pT172 (*green*) and pan-GAP-43 Abs (*red*). Magnified images of DIV 3 mouse hippocampal neuronal soma with several neurites. White boxes indicate ROIs that were measured. N1: longest neurite; N2: second longest neurite. Scale bar: 50 μm. **h** Means of the staining intensity ratios (pT172 vs. GAP-43) in neurites (of g; *rightmost*). Values are expressed as means ± SD; n = 10; Paired *t*-test; ***p < 0.001. Immunofluorescent experiments were performed using mAb against pT172

To characterize this phosphorylation site, we used an Ab specific to pT172 of GAP-43 and confirmed its reactivity and specificity (Additional file 2: Fig. S1a). The Ab recognized wild-type GAP-43 with T172, but not mutant GAP-43 in which T172 was changed to A, when expressed in Neuro 2A cells (Fig. 1a). pT172Ab also recognized growth cone particles prepared from developing rodent brain (Fig. 1b).

Next, we observed the distribution of pT172 in growth cones of cultured murine cortical neurons using super-resolution microscopy, which is a type of fluorescent microscopy that overcomes the optical diffraction limit (~ 200 nm) [15]. Devices for super-resolution microscopy are based upon different principles, and we used 3D-structured illumination microscopy (SIM) [15, 16].

The growth cone is divided into two domains: the C-domain is enriched in microtubules and vesicles, and the P-domain is rich in F-actin in lamellipodia and filopodia. Super-resolution microscopy showed that immunoreactivity with pT172Ab was distributed mainly in the C-domain, and partly in the P-domain (Fig. 1c, d). pT172 and the kinase that phosphorylates it, c-jun N-terminal kinase (JNK; [11–13, 17],see also Additional file 2: Fig. S1b), were colocalized in the growth cone in both the C- and P-domains (Fig. 1e). This immunoreactivity was also detected in the distal axon area and showed a gradient that was highest in the growth cone (Fig. 1f; see also Additional file 2: Fig. S1c). pT172Ab immunoreactivity in the growth cone was colocalized with GAP-43 (overlap coefficient: 0.64; Additional file 2: Fig. S1d); however, the former showed preferentially stronger immunoreactivity to the axon than the latter (Fig. 1g). Compared to the dendritic area, pT172 was concentrated in the axon (Fig. 1g, h).

### pT172Ab recognizes growing axons in the developing brain

**Fig. 2:**
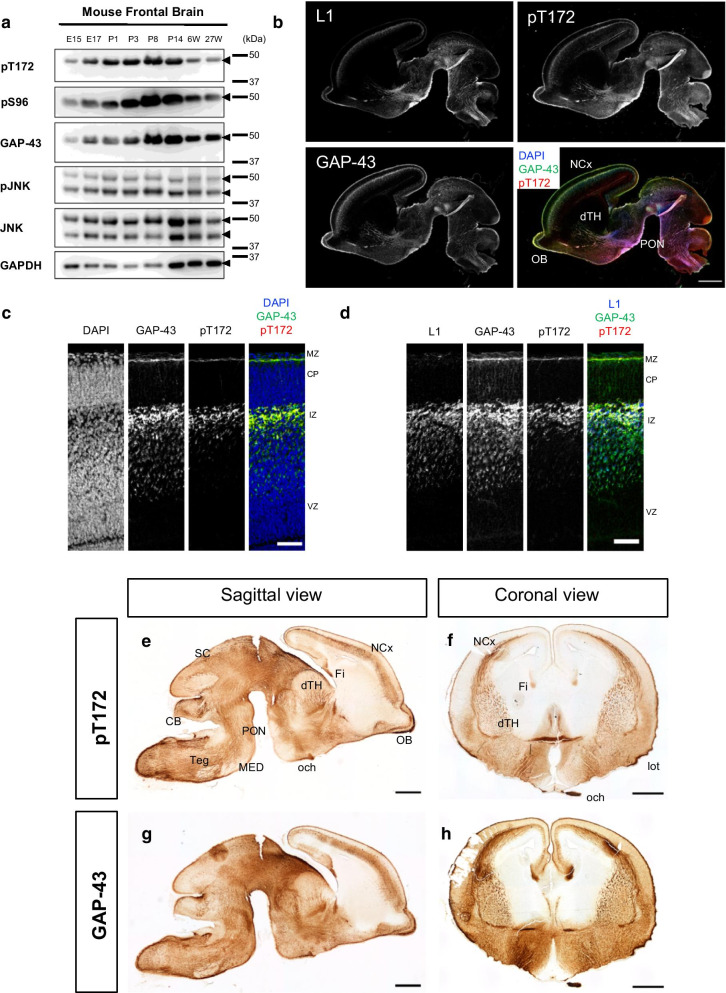
Expression patterns of GAP-43 and pT172 in developing mouse brain. **a** Immunoblotting of mouse frontal brain lysates. The expression of GAP-43 and pT172 persists throughout life. *Glyceraldehyde 3-phosphate dehydrogenase (GAPDH)*: positive control. The mouse strain was C57BL/6. **b** Immunohistochemistry of pT172, pan-GAP-43 and the cell adhesion molecule (L1), in E15 mouse brain in sagittal sections. Scale Bar; 500 μm. **c**, **d** Immunostaining of pT172Ab, GAP-43 Ab and co-staining of 4′,6-diamidino-2-phenylindole (DAPI) (**c**), or Immunostaining pT172, GAP-43 and L1 Abs (**d**). These views were obtained by Z-stack maximum-intensity projection fluorescence images of the neocortex. Cell body-specific nuclear staining with DAPI is shown. GAP-43 itself was expressed by migrating neurons and ingrowing axons in the intermediate zone (IZ). pT172 expression was restricted to the L1-positive thalamocortical axons in the upper IMZ. *MZ* marginal zone; *CP* cortical plate and *VZ* ventricular zone. Scale bar; 50 μm. **e**–**h** Spatial distribution pattern of pT172 (**e**, **f**) and GAP-43 (**g**, **h**) in E15 mouse brain. The immunoreactivity of GAP-43 was found in most of the differentiated neurons. In contrast, pT172 was distributed to the developing axonal processes, but was not detected in the neuronal cell bodies. *Fi* fimbria, *Och* optic chiasm, *OB* olfactory bulb, *sc* superior colliculus; *Teg* longitudinal tegmental tracts;* CB* cerebellum;* dTH* dorsal thalamus;* MED* medulla oblongata;* NCx* neocortex; and* PON* pons. Scale bars: 500 μm. Immunohistochemical studies (DAB staining) were performed using pAb against T172 (**e**–**h**). See also Additional file 4: Fig. S3

During mouse brain development, the amount of pT172 was elevated up to postnatal day (P)8, then decreased more rapidly than pS96 and GAP-43 itself (Fig. 2a, b; also see Additional file 3: Fig. S2a–c). pJNK, which is the activated form of JNK, is also similarly distributed during development [18], thus JNK is likely to phosphorylate this residue in the developing brain (see Fig. 1e).

Next, we performed immunohistochemistry on the developing mouse brain. On embryonic day (E)15, pT172Ab more selectively stained growing axons than the GAP-43 Ab itself (Fig. 2c, d); in particular, pT172 was restricted to the L1 (a cell adhesion molecule)-positive thalamocortical axons in the upper intermediate zone (IMZ; Fig. 2c, d; [12, 13]). pT172Ab also recognized growing axons in various regions (Fig. 2e–h; see also Additional file 4: Fig. S3a–i) rather than the cell bodies (Fig. 2c–h). Taken together, these results suggested that pT172Ab recognized most growing axons in the developing CNS just as well as pS96Ab [12, 13].

### pT172 is detected in regenerating axons

**Fig. 3:**
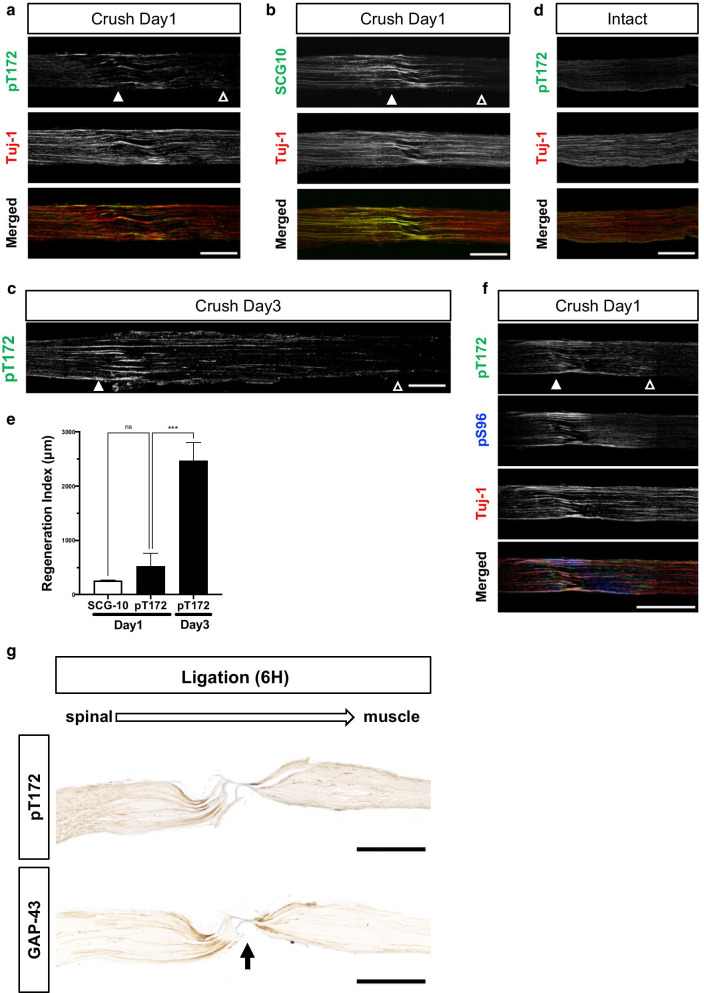
Axon regeneration of the injured sciatic nerve in adult mice is tightly associated with T172 phosphorylation of GAP-43. **a**–**c** Immunofluorescent studies of the sciatic nerves in longitudinal sections on days 1 (**a**, **b**) and 3 (**c**) after the crush using pT172, SCG-10, and Tuj-1 (neuron-specific β3 tubulin) Abs. SCG-10 was used as a positive control for axon regeneration. Both sections of (**a**) and (**b**) were adjacent with each other. **d** pT172 immunoreactivity was not detected in the contralateral intact side against (**a**, **b**). **e** Regeneration index [12, 13, 21] of pT172, compared to that of SCG10. The regeneration index on day 3 was higher than that on day 1. n = 3 (for each group). ***p = 0.0005 by one-way ANOVA with Bonferroni test. **f** The immunoreactivity of pT172 and pS96 on the sciatic nerve after the crush on day 1. *White arrowheads*: injury point; *black arrowheads*: the farthest point of positive immunoreactivity. Scale bar: 500 µm (**a**–**d**, **f**). Z-stack average-intensity projection images are shown (**a**–**d**, **f**). **g** Ligation of the sciatic nerves. In samples 6 h after ligation, pT172Ab immunoreactivity was stronger than in the proximal region than that in the distal one to the ligation site. *Arrow*: the ligation point. Scale bar: 500 μm. DAB staining was used for detection [12, 13, 17]. Ligation experiments were performed by method of Cavalli et al. [63]. See also Additional file 5: Fig. S4

Phospho-specific Abs (pS96Ab of GAP-43, pS25Ab of MAP1B, and pS1201Ab of MAP1B) also recognize regenerating axons in adult neural rodent tissues [12, 13, 17, 19]. Like the axon regeneration marker SCG10, immunohistochemistry with pT172Ab detected regenerating axons in the injured mouse sciatic nerve on days 1 and 3 ([20],Fig. 3a–c; see also Additional file 5: Fig. S4a, b). In the uninjured nerves, pT172Ab immunoreactivity was not detected, although that of GAP-43 itself was (Fig. 3d, see also Additional file 5: Fig. S4c). The regeneration index [21] of pT172 was also superior to that of SCG10 (Fig. 3e). Taken together, pT172 GAP-43 is associated with regenerating axons and is a marker for them [17, 22], similar to pS96 ([12, 13], see also Fig. 3f). Ligation experiments showed that pT172 was selectively positive in the region proximal to the ligature (Fig. 3g), suggesting that this phosphorylation was catalyzed in the cell bodies and that the phosphorylated form underwent anterograde axonal transport.

### pT172Ab recognizes growing axons of the developing ferret brain

**Fig. 4:**
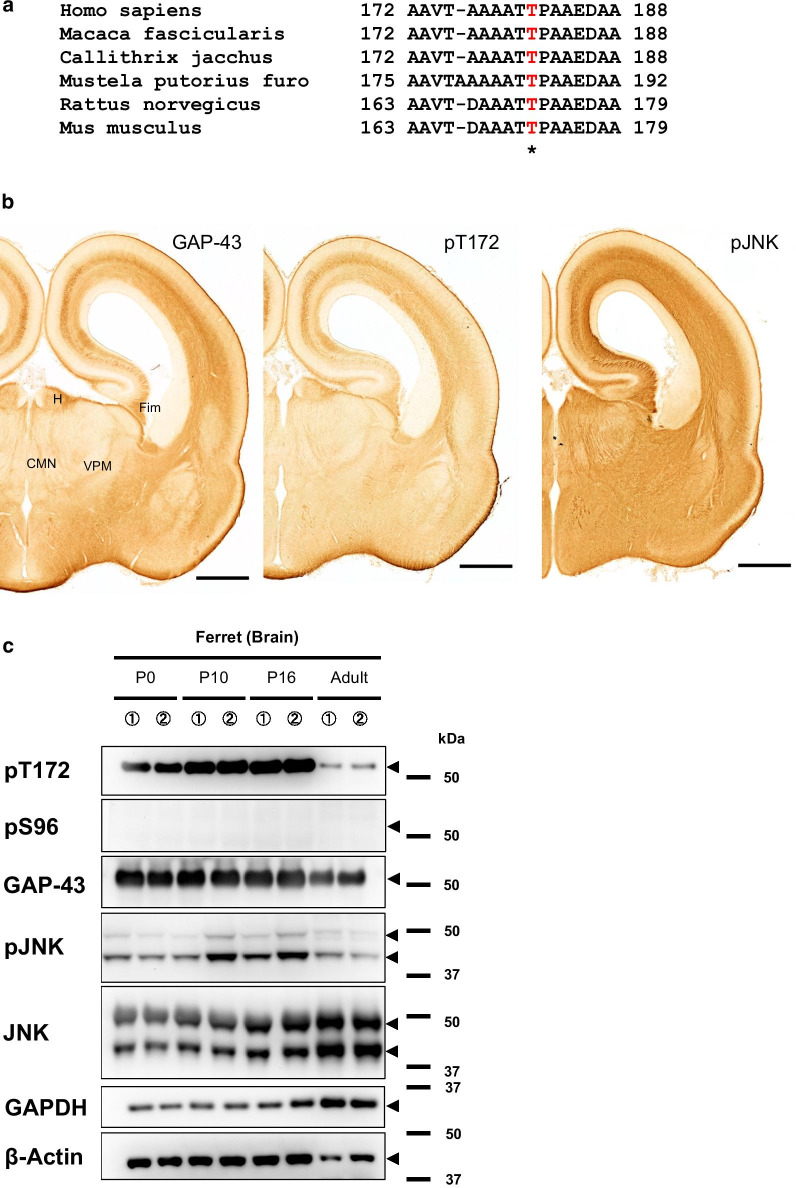
pT172 recognized growing axons in the ferret embryonic brain. **a** The pT172 site (*) and its surrounding amino acid sequences in GAP-43 are conserved from rodents to primates. Human (*H. sapiens*; 238 aa; T181), cynomolgus monkey (*Macaca fuscata*; 238 aa; T181), common marmoset (*Callithrix jacchus*; 177–186, 238 aa; T181), ferret (*Mustela putorius furo*; 242 aa; T185), rat (*Rattus norvegicus*; 226 aa; T172), and mouse (*Mus musculus*; 227 aa; T172). Note that the amino acid sequences of T172 and the following P173 in mouse are completely conserved in other mammals. **b** Immunohistochemistry (DAB staining) using pT172Ab in E40 ferret brain. Scale bar: 1 mm. **c** Patterns of immunoreactivity with pT172Ab in developing ferret cortices (P0, P10, P16, adult; duplicate samples). *GAPDH* and *β-actin*: positive controls. pT172 pAb was diluted to 1:1,000. *Arrowheads*: the predicted molecular mass. In the adult brain lysate, frontal lobes were used

Examination of the amino acid sequences shows that T172 in mouse GAP-43 and its neighboring sequences, in particular TP sequences, which are essential to the MAPK substrates, are completely conserved in rodents and higher mammals, suggesting that this site is a conserved phosphorylation site of GAP-43 (Fig. 4a; see also Additional file 6: Fig. S5).

Ferrets are a more advanced mammal than rodents, and also are used as a model animal in neuroscience [12, 13]. E40 ferret brain showed pT172Ab immunoreactivity in growing axons (Fig. 4b. In addition, the developmental patterns of the pT172Ab immunoreactivity in ferret (i.e., ferret pT185), was similar to that in mouse; phosphorylation was rapidly downregulated compared to GAP-43 itself (Fig. 4c). Taken together, in a wide range of mammals and not only rodents, GAP-43 pT172Ab potentially recognizes growing axons during the time course of development [23].

### GAP-43 in developing marmoset brain is recognized by pT172Ab

**Fig. 5:**
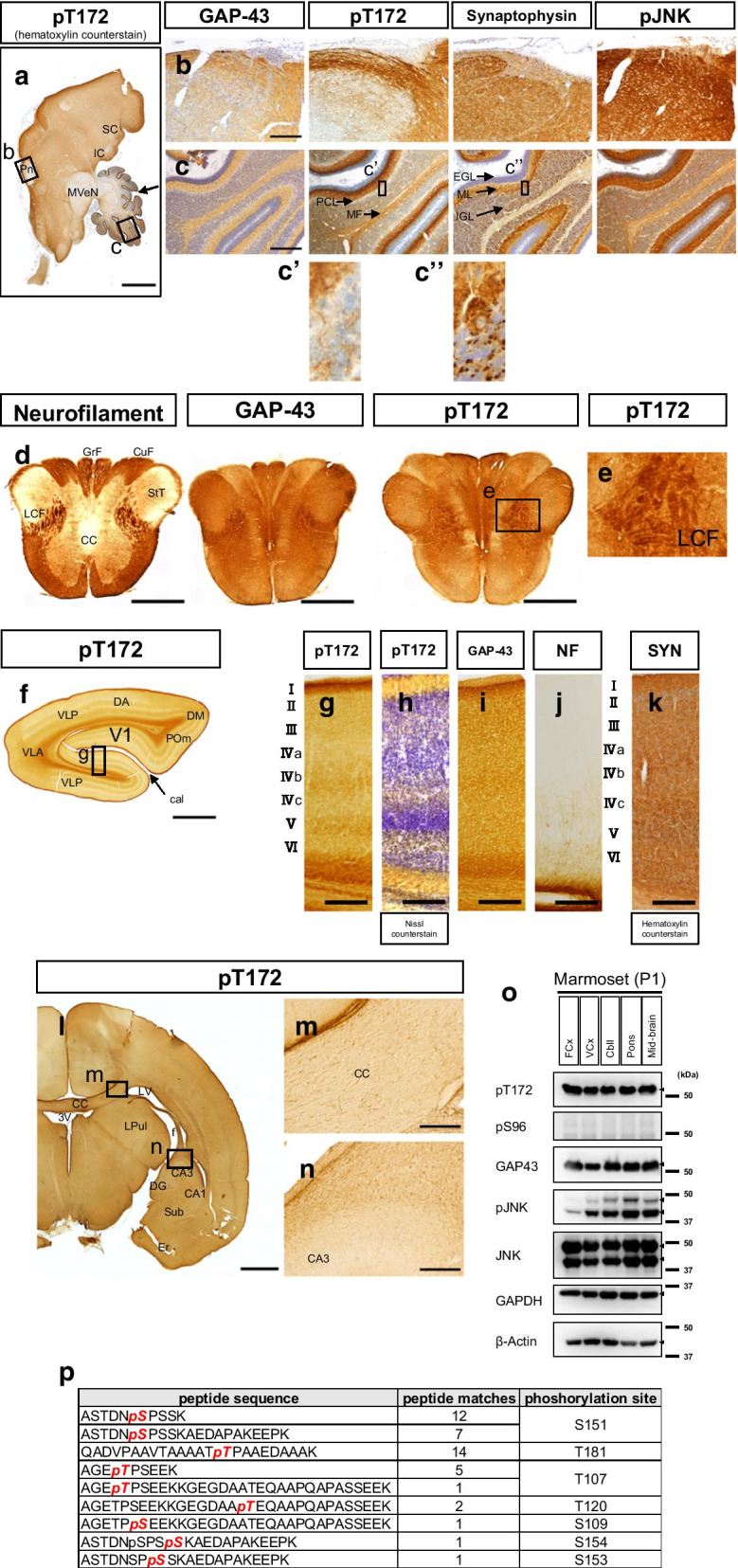
pT172Ab immunoreactivity in various regions of P1 marmoset brain. **a** pT172Ab immunoreactivity was detected in mid- and hind-brain together with hematoxylin counterstain. **b** and **c** Immunohistochemistry for pons (**b**) and cerebellum (**c**), respectively, using Abs against GAP-43, pT172, synaptophysin, and pJNK. Hematoxylin stained the neuronal nuclei as blue. Compared to GAP-43 itself and the synaptophysin Ab, pT172Ab immunoreactivity was selectively detected in the growing axons, not in the cell bodies. Phosphorylated JNK, its activated form, was colocalized with pT172Ab immunoreactivity in this tissue. Higher magnification images using pT172 (**c’**) and synaptophysin (**c’’**) Abs are shown from boxes in (**c**). pT172Ab immunoreactivity was not detected within Purkinje cells. *SC* superior colliculus; *IC* inferior colliculus; *MVeN* medial vestibular nucleus; *Pn* pontine nuclei. Primary fissures are noted with an arrow. Scale bar: 2000 μm in (**a**) and 200 μm in (**b**) and (**c**). *EGL* external molecular layer; *IGL* internal granule layer; *PCL* Purkinje cell layer; and *MF* mossy fiber. **d** pT172Ab immunoreactivity of the brain stem area and its enlargement (**e**). pT172Ab recognized in the elongating lateral corticospinal tract, which is also NF-M Ab stained. *CC* central canal; *CuF* cuneate fasciculus; *GrF* gracile fasciculus; *LCF* lateral corticospinal fiber; and *StT* spinal trigeminal tract. **f** pT172Ab immunoreactivity in the visual cortex. *Cal* calcarine sulcus; *DA* dorsoanterior extrastriate area;* DM* dorsomedial visual area; *POm* parietooccipital medial area; *V1* primary visual cortex; *VLA* ventriolateral anterior extrastriate area; and *VLP* ventriolateral posterior extrastriate area. Scale bar: 2000 μm. **g** Higher magnification images of box in (**f**). *I-VI*, the cortical layer numbers. pT172Ab immunoreactivity was observed in layers IVb and V. **h** Counterstaining using Nissl staining and pT172Ab. Because Nissl staining features the neuronal cell bodies, it suggests that pT172Ab immunoreactivity was not present there. Reference staining views in the adjacent sections are also shown as the immunoreactivity of (**i**) GAP-43Ab itself, **j** NF Ab, and **k** synaptophysin Ab (counterstained with hematoxylin). Scale bar: 200 μm (**g**–**k**). **l** pT172Ab immunoreactivity in the frontal cortex. Scale bar: 2000 µm. *CA* cornu ammonis; *CC* corpus callosum; *DG* dentate gyrus; *Er* entorhinal cortex; *f* fornix; *LV* lateral ventricle; *LPul* lateral pulvinar; *Sub*: subiculum; *3 V*: 3rd ventricle. **m**, **n** Higher magnification images of the box in (**l**). Scale bar: 200 µm. **o** Immunoblotting of several different regions of P1 marmoset brain using pT172Ab. *GAPDH* and *β-actin*: positive controls. *Arrowheads*: predicted bands. Note that the immunoreactivity of pJNKAb is generally associated with that of pT172Ab. *FCx* frontal cortex; *VCx* visual cortex; *Cbll* cerebellum; *Mid-brain* midbrain tissue partially containing pons. **p** Shotgun phosphoproteomics of a 50-kDa-level gel sample (see Additional file 7: Fig. S6c) of the P1 marmoset brain (midbrain containing pons) for GAP-43 phosphorylation sites. The detected phosphorylated peptides are shown in italic and bold letters

Common marmosets are currently used as a model primate in the neuroscience field [24, 25]. The aforementioned results suggest that GAP-43 in primates is phosphorylated at pT181 (T181 in primates corresponds to T172 in rodents) in their developing brains. pT172Ab immunoreactivity was observed both in the archicortex and the paleocortex of a P1 marmoset (Fig. 5a–c) and was observed primarily in the growing axons rather than in the cell bodies (Fig. 5b). Similarly, the same tendency was observed in the brainstem and the cerebellum (Fig. 5c–e; for example, the Purkinje cell layer was negative (Fig. 5c). pT172Ab showed high expression of pT181 of marmoset GAP-43 in the P1 marmoset neocortex (Fig. 5f, g). The immunoreactivity for pT172Ab was partly colocalized with neurofilament (NF) and synaptophysin (Fig. 5f–k). In the neocortex, brain development seemed to go forward at a further stage prior to that in the brainstem (Fig. 5l–n). pT172Ab immunoreactivity was not observed in the layers where the cell bodies were enriched (Fig. 5h). Its specific immunoreactivity was also confirmed in the marmoset brain (Fig. 5o; Additional file 7: Fig. S6a, b). In addition, this phosphorylation was generally associated with JNK activation in various brain regions (Fig. 5o). Direct phosphoproteomics of the P1 marmoset brain revealed that pT181 was one of the major phosphorylated sites in GAP-43 (Fig. 5p; see also Additional file 7: Fig. S6c).

### Human GAP-43 is phosphorylated by JNK, and its phosphorylation is recognized by pT172Ab

**Fig. 6:**
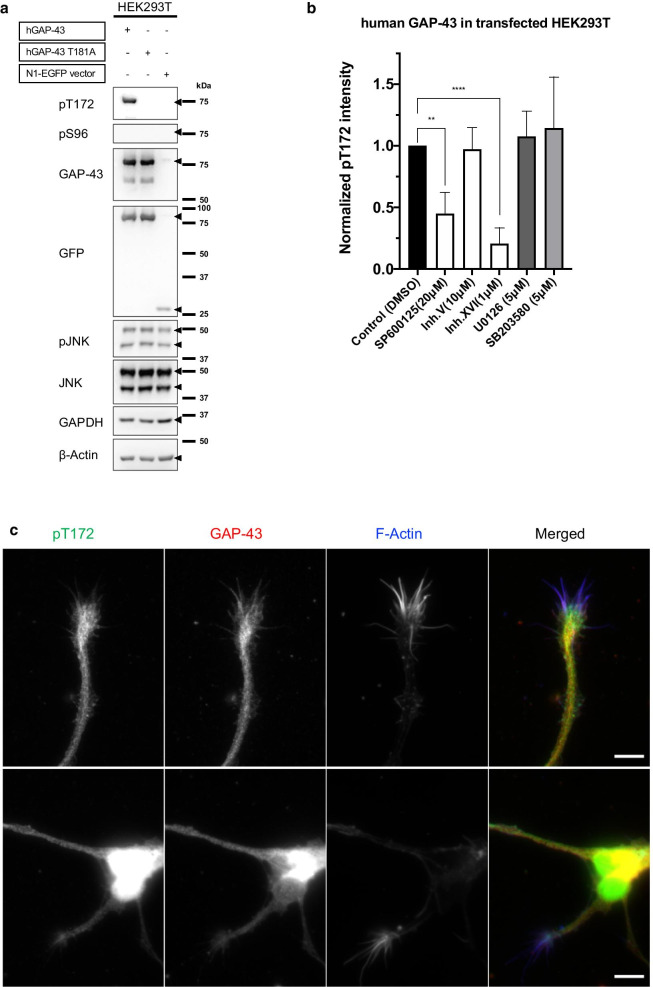
pT172Ab recognized human pT181 GAP-43, which corresponds to rodent pT172. **a** pT172Ab (*pT172*) reacted with expressed human GAP-43 in transfected HEK 293 T cells, as well as GAP-43 expressed from mouse cDNA (Fig. 1a). This reactivity is probably due to phosphorylation of T181 in human GAP-43 (see Additional file 6: Fig. S5). HEK 293T cells were transiently transfected with human GAP-43-EGFP, GAP-43 T181A-EGFP-tagged vector, or N1-EGFP (control vector). In each lane, 28 μg of protein was loaded. **b** Human GAP-43 T181 was phosphorylated by JNK. The pT172Ab reaction was blocked by inhibitors of JNK (SP600125 (20 μM), inh. V (10 μM), or inh. XVI (1 μM)) for 3 h. Inhibitors of other MAPK signaling pathways (MEK1/2 inhibitor, but specific as an ERK inhibitor at this concentration: U0126 (5 μM)) and p38 inhibitor: SP203520 (5 μM)) did not affect this phosphorylation. Normalized intensities were measured using pT172Ab, and as a control, the band intensity of untreated cell was used (0.88% DMSO as a control, equal to the volume of DMSO in SP600125). n = 5; means ± SD. One-way ANOVA followed by a post hoc test using the Bonferroni method. **p < 0.01; ****p < 0.0001. **c** pT172Ab recognized the growth cones and the axons of differentiated neurons derived from hiPSCs, as well as those of mouse (see Fig. 1c). pT172Ab immunoreactivity was colocalized with that of GAP-43 Ab. Scale bar: 30 μm

As shown in Fig. 4a, human GAP-43 T181 corresponds to T172 of rodent GAP-43. We thus examined whether this residue of human GAP-43 is phosphorylated. Transfection of HEK 293T cells with a human GAP-43-expressing vector revealed that this expressed GAP-43 was phosphorylated and recognized by pT172Ab (Fig. 6a), but the T181A mutant was not recognized by pT172Ab (Fig. 6a). T181 phosphorylation was specifically inhibited by JNK inhibitors such as SP600125 and inh. XVI, but not other MAPK inhibitors such those for p38 or ERK (Fig. 6b). In addition, we also confirmed that stress-dependent JNK activation [12, 13] phosphorylates pT181 (Additional file 8: Fig. S7). pT172Ab immunoreactivity was also enriched in distal axons (Fig. 6c). These results suggested that T181 of human GAP-43 is phosphorylated by JNK, similar to murine GAP-43.

## Discussion

GAP-43 is a vertebrate-specific neuronal protein that is widely thought to be related to axogenesis during brain development in a wide range of vertebrates [26–32], however, its involvement has not been directly demonstrated. For example, GAP-43-knockout mice show only slight abnormalities in neural development following neonatal death [33]. In this paper, we have shown that pT172 is concentrated in growing axons in a wide range of mammals, including rodents (Figs. 1 and 2), ferrets (Fig. 4), and primates (Figs. 5 and 6). pT172 itself was described more than a quarter century ago [34] just before the discovery of JNK [35], at which time its phosphorylation was not thought to be related to JNK.

### Phosphorylation of GAP-43 in relation to its structure

GAP-43 is an intrinsically disordered protein and thus does not have a specific tertiary structure and is believed to be very flexible [36, 37]. The structure is regulated by post-translational modifications such as phosphorylation [38, 39]. Such intrinsically disordered proteins, including histones, are highly phosphorylated [40], and the phosphorylation sites are thought to be important in its functions. Our phosphoproteomic studies revealed more than 20 different phospho-sites in GAP-43, including low-frequency sites [12, 13]. From fish to mammals, GAP-43 has more than one SP (serine-proline) or TP (threonine-proline) sequence in its intrinsically disordered regions, and these sites are likely to be a target of the high activity of JNK in the developing CNS. Although primate GAP-43 lacks the pS96 that is present rodents (Figs. 5p and 6a, the structural properties of pS96 and pT172 may not be largely different from each other. In rodents, these two phosphorylated sites are very similar to each other in the developing brain; they are tightly associated with growing axons or regenerating axons (Fig. 3f), and have similar distribution patterns in cultured growth cones [12, 13].

### Phosphorylation of GAP-43 in relation to protein kinases

The most frequently reported phospho-site of GAP-43 is S41 by protein kinase C (PKC) [41]. However, our phosphoproteomic analysis did not detect S41 in growth cone membranes or in regenerating axons of injured sciatic nerves [12, 13]. Previous immunohistochemistry for pS41 in the developing mouse brain [42] showed that pS41 is not likely to be colocalized with pS96 or pT172 in growing axons (Fig. 2, [12, 13]). Thus, at least for nerve growth or regeneration, we conclude that PKC phosphorylation of S41 is less important than S96 or T172 phosphorylation by JNK. Direct phosphoproteomics of marmoset brain also detected pT181, but not pS41 corresponding to the rodent S41 phosphorylation (Fig. 5p), supporting this idea. In developing neurons, an imaging study showed that Ca^2+^ mobilization is limited to only beneath the membrane [43]. Thus, PKC is probably not easily activated in growth cones as previously believed [44]. However, we cannot exclude the possibility that this phosphorylation in the adult brain is involved in events related to neuronal plasticity [45–48].

T172 and S96 are phosphorylated by JNK in rodent brains, and JNK is also activated in higher mammalian brains during axogenesis [49–54], review by [18]. JNK activation was at first thought to be mainly associated with apoptosis and cell survival [35, 55], however, within the last two decades, its high activity in developing rodent neurons has been observed during normal brain development at every step [22]. This JNK activation has not been demonstrated in organisms other than rodents,thus, our current results first showed the direct evidence (Fig. 6, b; Additional file 8: Fig. S7).

At this time, we do not know how and why JNK is activated in developing neurons during axon formation, although MKK7, a MAPK kinase that specifically activates JNK, is simultaneously activated [54]. JNK phosphorylates not only transcription factors such as c-jun, but also GAP-43 and MAP1B, as well as other components necessary for axon formation and growth [12, 13, 17]. Although GAP-43 is not expressed in invertebrates, and JNK-dependent sites in invertebrates are totally different from those of vertebrates [56], JNK activation is involved in normal axon growth even in *C. elegans* [57]. Considering our results in hiPSCs (Fig. 6c, d), from nematodes to humans, JNK activation is widely considered to be necessary for axon growth in developing neurons.

### Relationship between pS96 and pT172

JNK is a proline-directed protein kinase, and in the case of rodent GAP-43, three sites can be phosphorylated by this kinase, S96, S142, and T172 [12, 13], these sites are in fact phosphorylated in growth cone membranes. In human GAP-43, S106 corresponds to rodent S96, and the prior residue, P97, is conserved. However, K97 is inserted between S96 and P98, and thus, S96 is not phosphorylated because SP is disrupted; pS96Ab did not recognize ferret or human GAP-43 (Figs. 4c, 6a). These observations do not mean that pS96 is unimportant. pS96Ab and pT172Ab show quite similar patterns in the developing rodent brain, indicating that these two phosphorylation sites may have similar biological effects during nerve growth. In addition, we note that pT107, which is not detected in rodents due to this T position being substituted to A in rodents, was newly detected in the marmoset brain (Fig. 5p). Since this site is evolutionarily conserved in human and ferret GAP-43, pT107 in mammals other than rodents might have a similar role as pS96 in rodents.

### Roles of pT172 in developing and regenerating axons

Although pT172 in rodents or pT181 in more advanced mammals are markedly and specifically localized in developing axons, growth cones, and regenerating axons, the roles of this phosphorylation in these events remain unclear. GAP-43 itself has long been believed to regulate actin dynamics in the growth cone [45], however, this idea has not been recently revisited by the newest technology. In addition, we found that pT172 was localized in the distal axons in culture and in vivo during development (Figs. 1f and 2c–f). Therefore, the functions of pT172 do not seem to be confined to F-actin-rich areas (Additional file 2: Fig. S1c, d). pT172 appears to undergo anterograde axonal transport down to the regenerating axons (Fig. 3g), so in growing axons, pT172 might be involved in sorting or transport of other proteins necessary for axon formation.

Rapid downregulation of pT172 (Fig. 2a) or pT185 (Fig. 4c) is a feature of this type of phosphorylation. Probably, after synaptogenesis, this phosphorylation is inhibited although GAP-43 is still synthesized (Figs. 2a and 4c). At later stages of brain development, the frequency of actively growing axons where the levels of pT172 (mouse), pT181 (marmoset), or pT185 (ferret) are high, and that of stable axons where they are low, should be mixed. Neuronal networks likely form at distinct times in different brain regions of various mammalian species [23]. These factors are probably the reasons for the regional differences in pT172Ab immunoreactivity (Figs. 2, 4, 5, and Additional file 3: Fig. S2).

## Conclusion

Our phosphoproteomics analysis of rodent growth cone membranes showed that the highly frequent pT172 is widely conserved in growing axons and growth cones in various mammals. Because it is difficult to obtain marmoset embryos and early-stage ferret embryos, we cannot completely compare the most active stages of axon development in these animals [23] with rodents,however, in several regions of developing marmoset and ferret brains, the results are very similar to those of developing rodents (Figs. 2e–h, 4b, and 5). We found that pT172Ab was useful as a molecular marker for growing or regenerating axons, including those in primates. Such properties of pT172Ab will be very useful in current studies of human brain organoids derived from hiPSCs (cf. [58]).

As a next step, the molecular roles of this phosphorylated site in axon growth will be investigated in the developing brains of various species.

## Materials and methods

### Animals

All animal experiments were performed following approval from the Animal Resource Center of Niigata University, or from those of other institutions for marmosets (Nagoya City University) and ferrets (Kanazawa University). Pregnant and adult C57BL6N mice were obtained from Charles River Japan, Inc. (Atsugi Japan) and were used for neuronal cell culture, immunostaining of embryonic mouse brain and sciatic nerve injury, and analysis of protein expression and phosphorylation levels in developing brains.

### Abs used in experiments

Abs used for immunological detection in this paper and their dilutions are listed in Additional file 1: Table S1. As we previously described [12, 13], the polyclonal pT172Ab (pT172pAb) was produced by Sigma-Aldrich, for which phosphopeptide as an antigen (CVDAAATpTPAAED) was used, and monoclonal pT172 antibody (19-9A, pT172 mAb) was purchased from Fujifilm-WAKO.

### Studies of mouse or ferret brains at different stages

Mouse or ferret brains at several different stages were used for immunoblotting with pan-GAP-43 Ab or pT172Ab as described previously [17]. For murine developmental analysis of pT172, the whole brains of E14, E15, and E17, and the forebrains of P0, P8, and P14 adult mice (aged 6 W, 9 W, and 27 W) were homogenized in sample buffer with protease inhibitors and phosphatase inhibitors as described below. The brain lysates (20 μg protein) were loaded on SDS-PAGE electrophoresis gels. In the developmental analysis, the values were normalized to α-tubulin and compared to that of adult brain, which was defined as 1.0. To analyze the expression or the phosphorylation level of pT172, GeneTools analysis software (SYNGENE) was used to measure the densities of the immunoblotted bands.

### Plasmids, transfection, and inhibitor experiments

Inverse PCR-based mutagenesis techniques for the addition of a *myc* tag to the C-terminus of the mouse GAP-43 cDNA sequence by the pCMV-*myc* vector with a neuron-specific promoter [59] and for the addition of EGFP to the C-terminus of the human GAP-43 cDNA sequence (AM392759) by the pEGFP-N1 vector, which were purchased commercially, were performed using the KOD-Plus-Mutagenesis Kit (Toyobo Co., Ltd., Osaka, Japan). Neuro 2A cells were transfected with mouse GAP-43 plasmids by polyethyleneimine. HEK 293T cells were transfected with human GAP-43 plasmids using Lipofectamine 3000 (Thermo Fisher Scientific, Inc.) according to the manufacturer's instructions. These cells were collected for immunoblotting as described previously [12, 13, 17]. After 24–48 h, the transfected cells were homogenized using an ultra-sonicator in sample buffer (125 mM Tris–HCl pH 6.8, 20% glycerol, and 4% SDS) with protease inhibitors (20 μg/ml leupeptin, 10 μg/ml pepstatin, 1 mM phenylmethlsulfonyl fluoride, and 1 mM EDTA) and phosphatase inhibitors (1 mM NaF, 1 mM Na_2_MoO_4_, and 1 mM Na_3_VO_4_). For the inhibition assay of T172 phosphorylation, transfected HEK 293 T cells after a 24–48 h incubation were treated for 3 h with culture medium containing dimethylsulfoxide as a control, one of three JNK1 inhibitors [20 μM of SP600125 (Sigma-Aldrich), 10 μM of JNK Inhibitor V, or 1 μM of JNK Inhibitor XVI (Cayman Chemical, USA)], 5 μM of SB203580 (Sigma-Aldrich) as a p38 inhibitor, or 5 μM of U0126 (Cell Signaling Technology) as a MEK1/2 inhibitor that at this concentration works as an ERK inhibitor [12, 13, 17].

### Western blotting for analysis of GAP-43 phosphorylation levels

Western blotting procedures were previously described [12, 13]. Briefly, protein samples were separated by SDS-PAGE on a 10% gel, soaked in transfer buffer, and transferred to a PVDF membrane. The membrane was incubated with primary Abs overnight then with HRP-conjugated secondary Abs for 60 min at room temperature. Protein bands were visualized with an ECL Prime kit (GE Healthcare UK, Ltd.), and immunoblotting signals were acquired with a GENEGNOME Bio Imaging System (SYNGENE).

### Immunohistochemistry using mouse brains

Whole mouse brains at E15 were sectioned and used for immunohistochemistry with pT172Ab, GAP-43 Ab, pJNK Ab, and synaptophysin Ab as described previously [12, 13, 17]. In brief, E15 brains were fixed with 4% paraformaldehyde in 0.1 M phosphate buffer (PB, pH 7.4) for 24 h at 4 °C and cryoprotected with 30% sucrose in 0.1 M PB. Specimens were immersed in a solution consisting of OCT compound (Sakura Finetechnical Co., Ltd., Tokyo, Japan) and 30% sucrose/0.1 M PB and embedded by freezing in ethanol cooled with dry ice. Sagittal sections were sliced at a thickness of 20 μm using a sliding cryotome (CM1850; Leica Biosystems, Wetzlar, Germany) and thaw-mounted on MAS-coated slide glass (Matsunami Glass Inc., Ltd., Osaka, Japan). For diaminobenzidine staining, slides were incubated with methanol containing 0.3% H_2_O_2_ for 30 min, washed with 0.1–0.2% Triton X-100 in phosphate-buffered saline (PBS), and then incubated overnight with primary Abs diluted in 10% normal goat serum/0.1–0.2% Triton X-100 in PBS. The next day, the slides were reacted with *N*-Histofine Simple Stain Mouse MAX PO (R; Rabbit) (Nichirei Biosciences Inc., Tokyo, Japan), and a brown color was developed using a diaminobenzidine substrate kit (Nichirei Biosciences Inc.). Some multi-field images were acquired with a CCD camera (DP72, Olympus) and stitched with cellSens software (Olympus). The other multi-field images were obtained by a light microscope (BZ-800, KEYENCE Co., Japan).

For multiple fluorescent staining, sections were incubated with the primary Abs as described previously [12, 13], and then with Alexa Fluor 488-, 568-, or 647-conjugated secondary Abs (all from Invitrogen, USA). Fluorescence images were acquired with a confocal microscope (FV1200, Olympus).

### Immunohistochemistry using ferret and marmoset brains

Immunohistochemistry with E40 ferret brain was performed as described previously [60, 61]. Ferret brains were cut at 40-μm thicknesses using a sliding microtome.

P1 marmoset brains were fixed and immunostained as described previously [62]. We used common marmosets to which 5-ethynyl-2′-deoxyuridine (EdU) was administered for the purpose of labeling proliferating cells as previously described [24]. Marmosets were deeply anesthetized with isoflurane and transcardially perfused with PBS followed by PFA in 0.1 M PB.

The marmoset brains were extracted and post-fixed with the same fixative for 24–48 h. Then they were cryoprotected in 30% sucrose/0.1 M PB for 2–3 days. The samples were cut at 50-μm thicknesses (Retratome REM-710 & MC-802A Electro freeze, Yamato Kohki Industrial, Saitama, Japan). The free-floating sections were incubated with methanol containing 0.3% H_2_O_2_ for 30 min, washed three times with 0.1–0.2% Triton X-100 in PBS (PBST), blocked with 10% normal goat serum (NGS) in PBST or 5% skim milk in PBST for 30 min, and incubated with primary Abs diluted in blocking solution overnight at 4 °C. For diaminobenzidine staining, sections were washed three times with PBST, the sections were reacted with an N-Histofine Simple Stain MAX PO (R) kit, the brown color was developed with a diaminobenzidine substrate kit (Nichirei Co., Japan), and then sections were thaw-mounted on glass slides. The sections were then dehydrated with ethanol, rendered transparent with xylene, and finally mounted in Excel mount (Falma, Japan). In some cases, sections were further stained with Mayer’s hematoxylin solution (WAKO, Japan) or crystal violet (Sigma-Aldrich) for Nissl staining. DAB-stained samples were observed through an upright microscope (BX63, Olympus, Japan).

### Sciatic nerve injury experiments

Sciatic nerve crush injury of anesthetized adult C57BL/6 mice (17–31 weeks old) was performed using forceps crush surgery as described previously [12, 13, 17]. Sciatic nerve ligation experiments were performed as described previously [63] with minor modifications. The sciatic nerve on one side was crushed, and that on the other side was sham-operated as the negative control. One and three days after crush injury experiments and 6 h after ligation experiments, the mice were perfused intracardially with PBS followed by 4% paraformaldehyde. Then, the nerves were washed with PBS, immersed in 0.1 M phosphate buffer with 20% sucrose for an additional 1–2 days, and cut into 20- to 25-μm-thick longitudinal sections. Sciatic nerve samples were immunostained using the same procedure as described above.

### Super-resolution and confocal microscopic observation of cultured neurons immunostained using pT172Ab

Mouse E15 cortical or hippocampal neurons were differentiated into stage 3, when each neuron has an axon [64], and immunofluorescence with pT172Ab and observation of these neurons using 3D-SIM super-resolution were performed as described previously [16]. Immunofluorescence intensity was quantified as described previously [12, 13]. In brief, 3–7 day in vitro mouse hippocampal and cortical neurons were cultured in neurobasal medium (Gibco) with B-27 (Gibco), GlutaMax (Gibco), and antibiotics (penicillin–streptomycin, Sigma). Cells on glass coverslips were fixed with 1% glutaraldehyde (Sigma) in the medium and treated with 1% tetrahydroborate in cytoskeleton stabilizing buffer (CSB: 137 mM NaCl, 1.1 mM Na_2_HPO_4_, 5 mM KCl, 0.4 mM KH_2_PO_4_, 4 mM NaHCO_3_, 2 mM MgCl_2_, 5.5 mM glucose, 2 mM EGTA, and 5 mM PIPES [pH 6.1]) for reducing background staining for 10 min. They were then permeabilized with 0.1% Triton X-100 (Nacalai Tesque) in CSB for 10 min, were blocked with 3% bovine serum albumin (BSA, Nacalai Tesque) for 30 min, and reacted with primary and secondary Abs in 3% BSA. To visualize F-actin, the cells were incubated with rhodamine-phalloidin (1:500; Sigma-Aldrich). Quantitative distribution of the region of interest (ROI) of the growth cone area was measured by fluorescence intensity along a line plot from the filopodial tip to display a two-dimensional graph of pixel intensity for pT172 and F-actin images using ImageJ software Fiji (http://rsb.info.nih.gov/ij). The x-axis represents the distance along the line, and the y-axis is the normalized gray value.

Fluorescent images were acquired with a confocal laser scanning microscope (FV1200, Olympus) as previously described [12, 13]. For line graph analysis of axons, the fluorescence intensity of pT172 was selected from the longest neurite as the ROI, and the fluorescence value from the cell body toward the growth cone was obtained as the gray value per pixel.

The ratio of the fluorescence values of GAP-43 and pT172 in the longest neurite defined as an axon and the second longest neurite defined as a dendrite was obtained using Fiji. The average fluorescence intensity of each ROI was measured.

### Immunofluorescent studies of neurons that were differentiated from hiPSCs

Human induced pluripotent stem cells (1210B2), which were established from peripheral blood mononuclear cells (ePBMCs®, Cellular Technology Limited, OH, USA) at the Center for iPS Cell Research and Application, Kyoto University (CiRA: Kyoto, Japan) by an integration-free reprogramming method, were induced into neural progenitor cells using the dual SMAD-inhibition method with dorsomorphin plus SB431542 as previously described [65, 66]. hiPSCs were differentiated into stage 3 neurons [64, 67], then fixed with 4% PFA and 0.1% glutaraldehyde in 0.1 M PBS for 15 min at room temperature, and immunostained as described previously [68]. For visualization of F-actin, cells were incubated with rhodamine-phalloidin (1:500; Molecular Probes, Eugene, OR). Images were obtained by a CCD Camera (CoolSNAP HQ2, Photometrics).

### LC/MS/MS of marmoset brains

The shotgun proteomics method was described previously [12, 13]. Briefly, the protein extract from marmoset brain (midbrain containing pons) was prepared as described above. Two equal aliquots of protein extract (50 μg) were separated in parallel on a polyacrylamide gel (ANK KD, Mini-PROTEAN TGX,Bio-Rad Laboratories) and stained with Coomassie Brilliant Blue R-250. The two gel portions containing GAP-43, as indicated by immunoreactivity with anti-pan-GAP-43 Ab (D9C8, CST), were manually excised and collected in a low-binding microcentrifuge tube for in-gel trypsin digestion.

The gel slices were reduced with 10 mM dithiothreitol, carbamide methylated with 55 mM iodoacetamide, and subjected to in-gel trypsin digestion to improve recovery of the digested peptides. The peptides from each sample were finally dissolved in 15 μl of 0.3% trifluoroacetic acid. A 50-kDa-level gel sample (3 μl of 1/5 dilute solution) and a 10-kDa-level gel sample (4 μl) were injected into a nano-flow-LC (Eksigent nanoLC 415 with ekspert cHiPLC; Sciex) coupled with a tandem MS (TripleTOF5600; Sciex).

Analysis was conducted in triplicate for each sample under direct injection mode with an analytical column of 75 μm × 150 mm (Nikkyo Technos Co., Ltd.). Mobile phases A and B were 0.1% formic acid and 0.1% formic acid in acetonitrile, respectively. Peptides were eluted using 40 (10 kDa) or 60 (50 kDa)-min gradients from 2 to 32% B at 300 nl/min. MS spectra (250 ms) followed by 10 MS/MS spectra (100 ms each) were acquired in data-dependent mode. The dynamic exclusion time was set at 5 s. Autocalibration using 50 fmol of tryptic peptides of bovine serum albumin was performed. Protein identification was carried out using Mascot version 2.6.0 (Matrix Science) as a search engine (see Additional file 7: Fig. S6c). The peptide and MS/MS tolerance were set at ± 20 ppm and ± 0.1 Da, respectively. Modification settings were: fixed modification, carbamidomethylation of cysteine, variable modifications, deamidation of asparagine and/or glutamine, phosphorylation of serine and/or threonine, N-terminal glutamine to pyroglutamate, N-terminal glutamate to pyroglutamate, and oxidation of methionine. A maximum of two missed cleavages was allowed. The significance threshold was set at p < 0.05, which gave a false discovery rate (FDR) of < 0.05 for all identification results. Only peptides with a score exceeding the “Identity threshold” were employed. The GAP-43 protein coverage and peptide identification rate were 85%. Peptide matches: the number of peptides matched to GAP-43 with a Mascot score larger than the identity threshold and an FDR < 5% (see Fig. 5p).

### Multiple sequence alignment analysis

The National Library of Medicine (NCBI) protein database was used to search for amino acid sequences in human, cynomolgus monkey, marmoset, ferret, rat, and mouse GAP-43. The GAP-43 protein sequence of ferret was that of the predicted one. Multiple sequence alignment analysis was displayed using these search results by Snap gene software (Version 5.1.7; GSL Biotech).

### Statistics

All data are shown as the means ± standard deviation (SD). Paired Student’s *t* test and one-way analysis of variance (ANOVA) were used for statistical analysis, as appropriate, and a p-value < 0.05 was considered statistically significant. Significance testing using one-way ANOVA was further performed followed by a post hoc test using Bonferroni methods. All statistical analyses were performed using GraphPad Prism 8 software.

## Supplementary Information

**Additional file 1: Table 1.** Antibodies used for immunological detection in this paper.

**Additional file 2: Figure S1.** Intracellular localization of phosphorylated GAP-43 T172 and its responsible kinase. (a) As well as pan-GAP-43 Ab (left), the immunoreactivity of pT172 Ab on full-size membranes (right), showing a wider range of molecular masses, indicated that this Ab mostly recognizes a single band of mouse GAP-43 (mGAP-43) in the total proteins derived from the lysate of cultured mouse cortical neurons (DIV3). (b) In vitro phosphorylation of GAP-43 by activated JNK. Myc-GAP-43 was immunoprecipitaed using anti-myc and used as the JNK substrate. Phosphorylation experiments were performed following the manufacturer’s manual of “JNK1, Acitve” (M33-10g, SignalChem). (c) Immunostaining of cultured hippocampal neurons using pT172 Ab (blue) with rhodamine-phalloidin (red) and Tuj-1 Ab (green). Note that pT172Ab labeled the tubulin-positive area of the axon more intensely than the actin-positive area. Scale bar: 20 μm. (d) pT172Ab immunoreactivity in the growth cone was colocalized with GAP-43 (overlap coefficient: 0.62, calculated by ZEISS ZEN software) at ROI (white box). Scale bar: 10 μm.

**Additional file 3: Figure S2**. pT172 and GAP-43 are regulated in mouse developing brain. (a) The distribution of GAP-43, pS96, and pT172 persists throughout life (E14, P3, P8, P14, and 9W; 18.75 g of proteins in each lane). GAPDH: positive control. The frontal cortical lysates of a C57BL6N mouse were used. (b) Densitometric quantitation of (a). Values are represented as the means ± SD for three experiments. Although some GAP-43 expression was maintained throughout life, the immunoreactivity of pT172 and pS96 decreased rapidly with development. One-way ANOVA followed by a post hoc test using the Bonferroni method. *p < 0.05; **p < 0.01; ***p < 0.001; ****p < 0.0001. (c) Immunoblot for pT172Ab on full-size membranes. Note that this Ab mostly recognizes a single band of GAP-43 in the total protein of cortical lysate.

**Additional file 4: Figure S3.**. Expression pattern of pT172 in the developing mouse brain. (a) Negative control immunostaining of E15 thalamocortical pathway. pT172 pAb (a-1), secondary Ab alone (a-2; goat anti-rabbit Ab), and nonspecific IgG (a-3; rabbit serum). Each concentration of proteins was 0.26 µg/ml. Scale bar: 200 µm. (b) Microscopic images of sagittal sections derived from various brain regions were DAB-stained using pT172. Boxes in (b) represent the regions enlarged in (c-g). pT172 succeeded in labeling nerve fibers under a growing state. Scale bar: 500 µm in (b), 200 µm in (c; in c-g). (h, i) Z-stack maximum-intensity projection images of basal nuclei (h) and OB (i). pT172 was more closely colocalized in the developing axons labeled by L1 than pan-GAP-43 in OC, LOT, and STR. GAP-43 and pT172 were detected in neurons of MCL and ONL (i). CP: cortical plate; LOT: longitudinal olfactory tract; MCL: mitral cell layer; OB: olfactory bulb; OCh: optic chiasm; ONL: olfactory nerve layer; Teg: longitudinal tegmental tracts; STR: striatum; PON: pons; EGL: external granular layer, PK: Purkinje cell layer.

**Additional file 5: Figure S4.** pT172Ab immunoreactivity in regenerating axons after neural tissue injury in young adult mice. (a, b) pT172Ab immunoreactivity detected in injured sciatic nerve (day 1 (a) and day 3 (b) after crush injury). Black-arrowhead: damaged point; white-arrowhead: the farthest point of immunoreactivity. (c) pT172Ab immunoreactivity was not present in uninjured sciatic nerves, while GAP-43 reactivity itself was detected there. Scale bar: 500 μm. (a-c). See also Fig. 3.

**Additional file 6: Figure S5.** Amino acid sequences of GAP-43 surrounding the residue corresponding to S96 of rodents, in various mammalian species. S96 position of rodent GAP-43 is shown in red. See Discussion.

**Additional file 7: Figure S6.** Phospho-specificity of GAP-43 pT181 in the P1 marmoset. (a) Negative control immunostaining of P1 Marmoset visual cortex. pT172 pAb (a-1), secondary Ab alone (a-2; goat anti-rabbit Ab), and nonspecific IgG (a-3; rabbit serum). See also Fig. S3. Scale bar; 500 µm. (b) Immunoblot reactivity of pT172 Ab on a full-sized blot membrane. These results show the specificity of pT172Ab. (c) Phosphoproteomics procedure for the mid-brain including pons of P1 marmoset. The band corresponding to GAP-43 was cut out after SDS-PAGE of the sample, trypsinized in-gel, and subjected to MS analysis. *, molecular mass marker. Note that the GAP-43 immunoreactivity band (arrow in red) did not consist of the GAP-43 amino acid sequence by LC/MS/MS analysis, suggesting that it may be a non-specific reaction. The analyzed results are shown in Fig. 5p.

**Additional file 8: Figure S7.** Stress-dependent JNK activation phosphorylates human GAP-43 T181 and is recognized by pT172Ab. Extracts from the transfected U251G cells were prepared at 30 min after osmotic stress (0.5 M NaCl) for JNK activation and immunoblotted using pT172, GAP-43, GFP, pJNK, JNK, and GAPDH Abs by the methods of Kawasaki et al. (2018).

## Data Availability

Data sharing not applicable to this article as no datasets were generated or analyzed during the current study.
