# Supplementary material for: Phosphorylation of GAP-43 T172 is a molecular marker of growing axons in a wide range of mammals including primates
Source: Mol Brain. 2021 Apr 8;14:66. doi: 10.1186/s13041-021-00755-0 (PMC8034164; doi:10.1186/s13041-021-00755-0)
Supplement: Supplementary file 1 — Additional file 1: Table 1. Antibodies used for immunological detection in this paper. [file 13041_2021_755_MOESM1_ESM.docx]

**Table S1 Antibodies used for immunological detection in this paper**

| **Antibody** | **Supplier** | **Species** | **Dilution** | | |
| --- | --- | --- | --- | --- | --- |
|  |  |  | **WB** | **IF** | **IHC** |
| GAP-43 (pan) (AB5220) | Millipore | rabbit | 1:1000 | - | 1:1000 |
| GAP-43 (pan) (Af500) | Frontier Institute co., ltd | guinea pig | - | (C)1:100,(T)1:500 | - |
| GAP-43 (pan) (D9C8) | Cell Signaling technology | rabbit | 1:1000 | (T)1:500 | 1:1000 |
| GAP-43 (pT172) | Our group / Sigma-Aldrich | rabbit | 1:1000 | (C)1:100,(T)1:500 | 1:1000 |
| GAP-43 (pT172) (19-9A) | Our group / FUJIFILM WAKO | mouse | - | (C)1:100 | - |
| GAP-43 (pS96) (18-10H-9H) | Our group / FUJIFILM WAKO | mouse | 1:1000 | (T)1:500 | - |
| L1CAM (clone 324) (MAB5272) | Merck | rat | − | (T)1:100 | - |
| pJNK (#4668) | Cell Signaling technology | rabbit | 1:500 | (C)1:100 | 1:100 |
| JNK (#9252) | Cell Signaling technology | rabbit | 1:1000 | - | - |
| Neurofilament M (NF-M) (2H3) | Developmental Hybridoma Bank | mouse | - | - | 1:1000 |
| Phosphorylated Neurofilament | Sternberger Monoclonals | mouse | - | (C)1:10000 | − |
| Synaptophysin (clone 27G12) | Leica biosystems | mouse | - | - | 1:100 |
| β-tubulin III Biotinylated (TUJ1) | R&D systems | mouse | - | (C)1:100,(T)1:500 | - |
| GAPDH mAb-HRP-DirecT | MBL JAPAN | mouse | 1:2000 | - | - |
| β-Actin pAb-HRP-DirecT | MBL JAPAN | rabbit | 1:2000 | - | - |
| α-tubulin pAb-HRP-DirecT | MBL JAPAN | rabbit | 1:2000 | - | - |
| GFP pAb-HRP-DirecT (598-7) | MBL JAPAN | rabbit | 1:2000 | - | - |

WB: Western blotting; IF: Immunofluorescence; IHC: Immunohistochemistry; C: cell staining; T: tissue staining
