# Supplementary figures and images for: Phosphorylation of GAP-43 T172 is a molecular marker of growing axons in a wide range of mammals including primates
Source: Mol Brain. 2021 Apr 8;14:66. doi: 10.1186/s13041-021-00755-0 (PMC8034164; doi:10.1186/s13041-021-00755-0)

## Slide 1
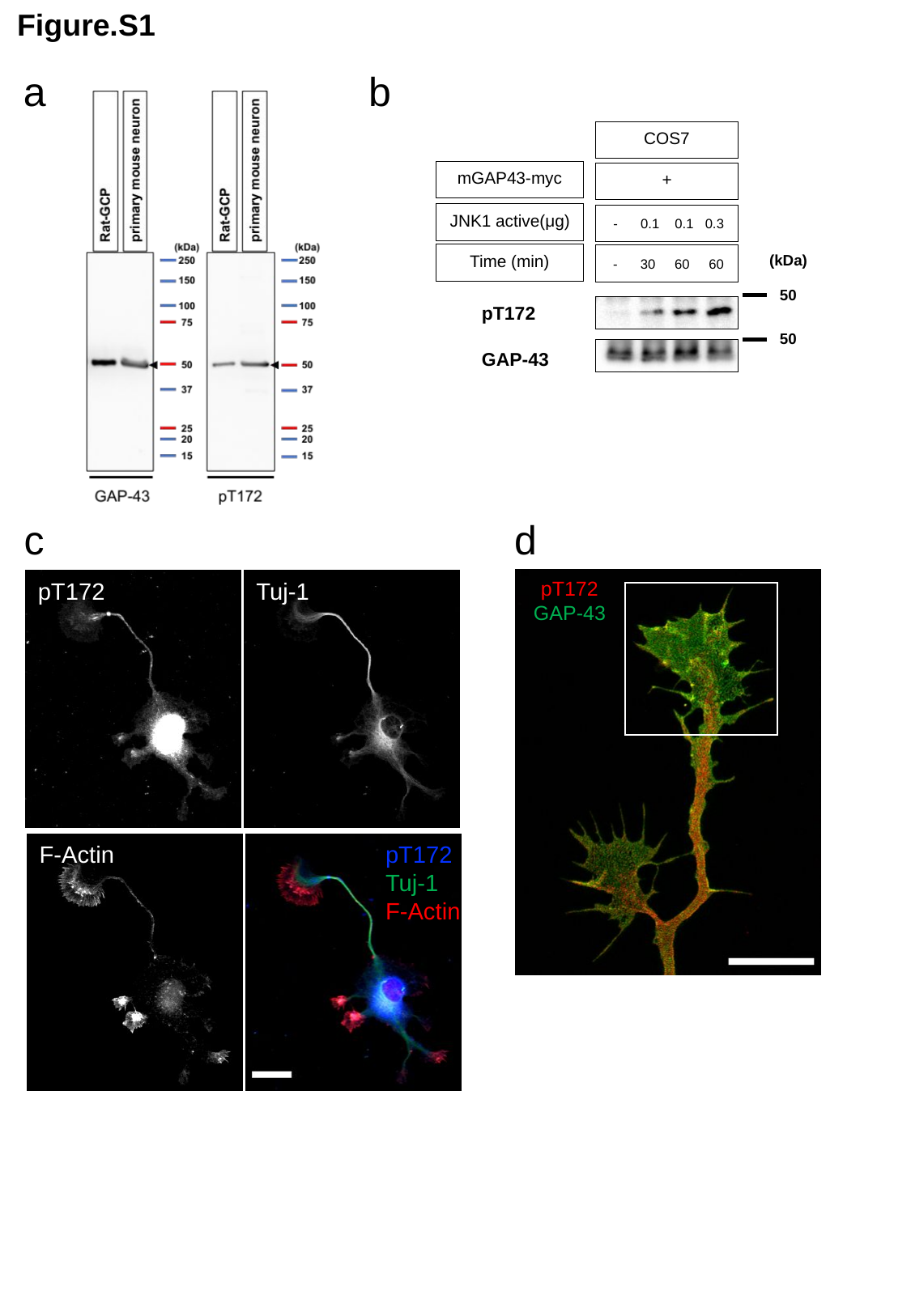

Figure.S1
a
b
COS7
mGAP43-myc
+
JNK1 active(μg)
 - 0.1 0.1 0.3
Time (min)
(kDa)
 - 30 60 60
50
pT172
50
GAP-43
c
d
pT172
GAP-43
pT172
Tuj-1
F-Actin
pT172
Tuj-1
F-Actin

Supplement: Supplementary file 2 — Additional file 2: Figure S1. Intracellular localization of phosphorylated GAP-43 T172 and its responsible kinase. (a) As well as pan-GAP-43 Ab (left), the immunoreactivity of pT172 Ab on full-size membranes (right), showing a wider range of molecular masses, indicated that this Ab mostly recognizes a single band of mouse GAP-43 (mGAP-43) in the total proteins derived from the lysate of cultured mouse cortical neurons (DIV3). (b) In vitro phosphorylation of GAP-43 by activated JNK. Myc-GAP-43 was immunoprecipitaed using anti-myc and used as the JNK substrate. Phosphorylation experiments were performed following the manufacturer’s manual of “JNK1, Acitve” (M33-10g, SignalChem). (c) Immunostaining of cultured hippocampal neurons using pT172 Ab (blue) with rhodamine-phalloidin (red) and Tuj-1 Ab (green). Note that pT172Ab labeled the tubulin-positive area of the axon more intensely than the actin-positive area. Scale bar: 20 μm. (d) pT172Ab immunoreactivity in the growth cone was colocalized with GAP-43 (overlap coefficient: 0.62, calculated by ZEISS ZEN software) at ROI (white box). Scale bar: 10 μm. [file 13041_2021_755_MOESM2_ESM.pptx]

Figure S2

a

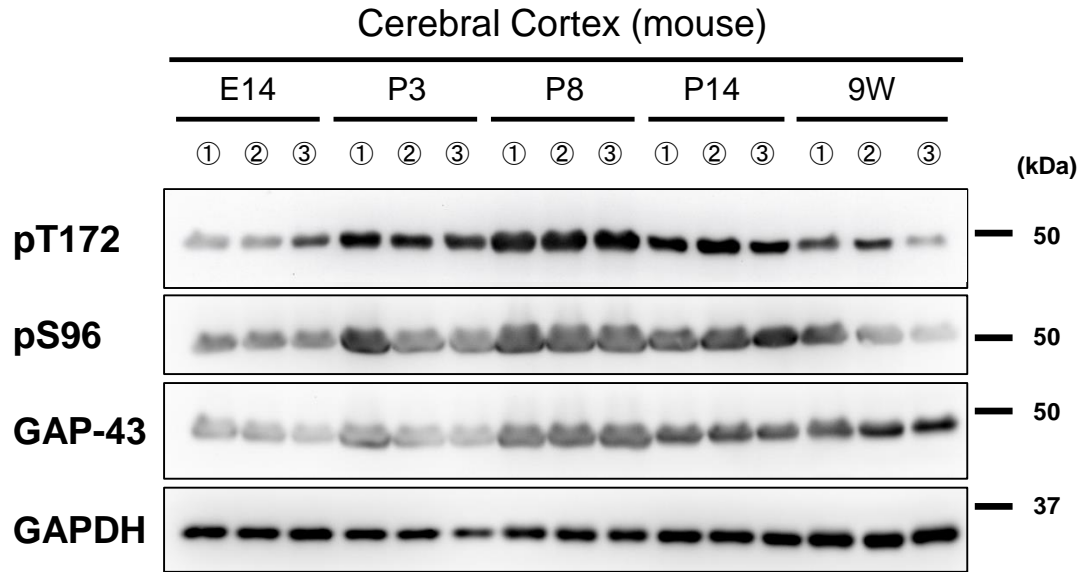

b-1

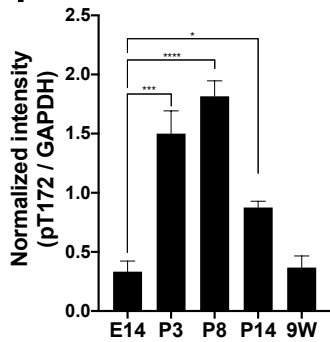

b-2

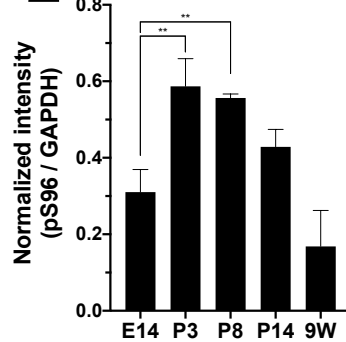

b-3

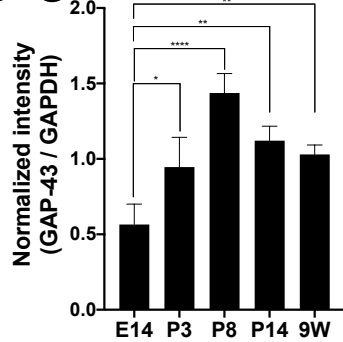

C

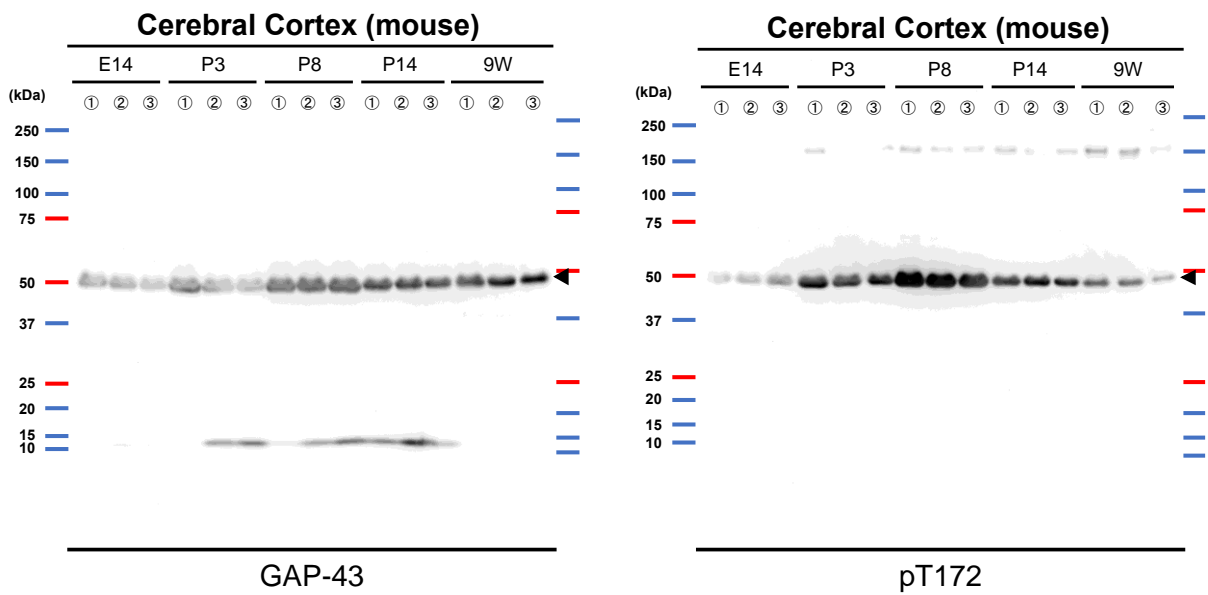

Supplement: Supplementary file 3 — Additional file 3: Figure S2. pT172 and GAP-43 are regulated in mouse developing brain. (a) The distribution of GAP-43, pS96, and pT172 persists throughout life (E14, P3, P8, P14, and 9W; 18.75 g of proteins in each lane). GAPDH: positive control. The frontal cortical lysates of a C57BL6N mouse were used. (b) Densitometric quantitation of (a). Values are represented as the means ± SD for three experiments. Although some GAP-43 expression was maintained throughout life, the immunoreactivity of pT172 and pS96 decreased rapidly with development. One-way ANOVA followed by a post hoc test using the Bonferroni method. *p < 0.05; **p < 0.01; ***p < 0.001; ****p < 0.0001. (c) Immunoblot for pT172Ab on full-size membranes. Note that this Ab mostly recognizes a single band of GAP-43 in the total protein of cortical lysate. [file 13041_2021_755_MOESM3_ESM.pdf]

## Slide 1
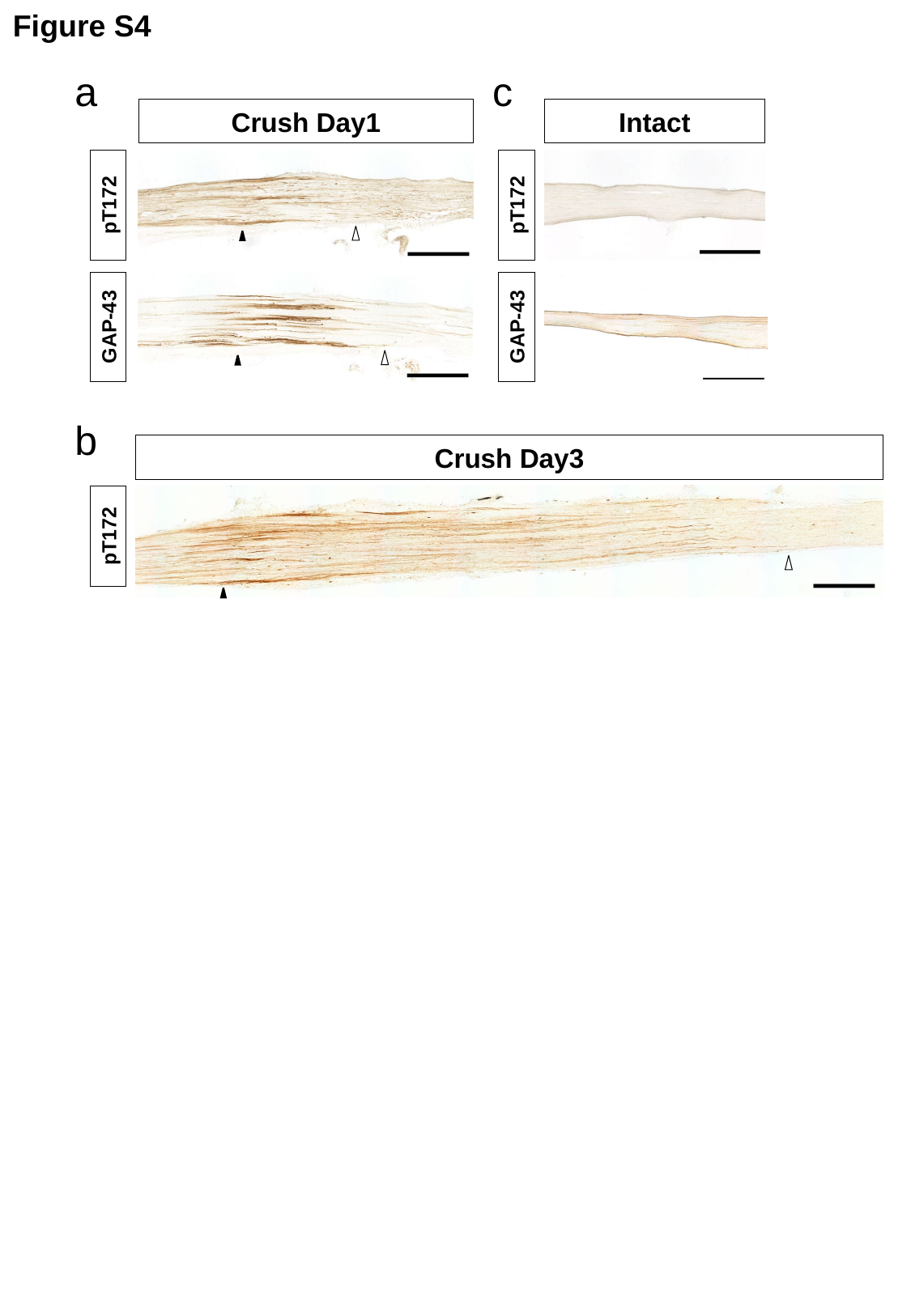

Figure S4
a
c
Crush Day1
Intact
pT172
pT172
GAP-43
GAP-43
b
Crush Day3
pT172

Supplement: Supplementary file 5 — Additional file 5: Figure S4. pT172Ab immunoreactivity in regenerating axons after neural tissue injury in young adult mice. (a, b) pT172Ab immunoreactivity detected in injured sciatic nerve (day 1 (a) and day 3 (b) after crush injury). Black-arrowhead: damaged point; white-arrowhead: the farthest point of immunoreactivity. (c) pT172Ab immunoreactivity was not present in uninjured sciatic nerves, while GAP-43 reactivity itself was detected there. Scale bar: 500 μm. (a-c). See also Fig. 3. [file 13041_2021_755_MOESM5_ESM.pptx]

## Slide 1
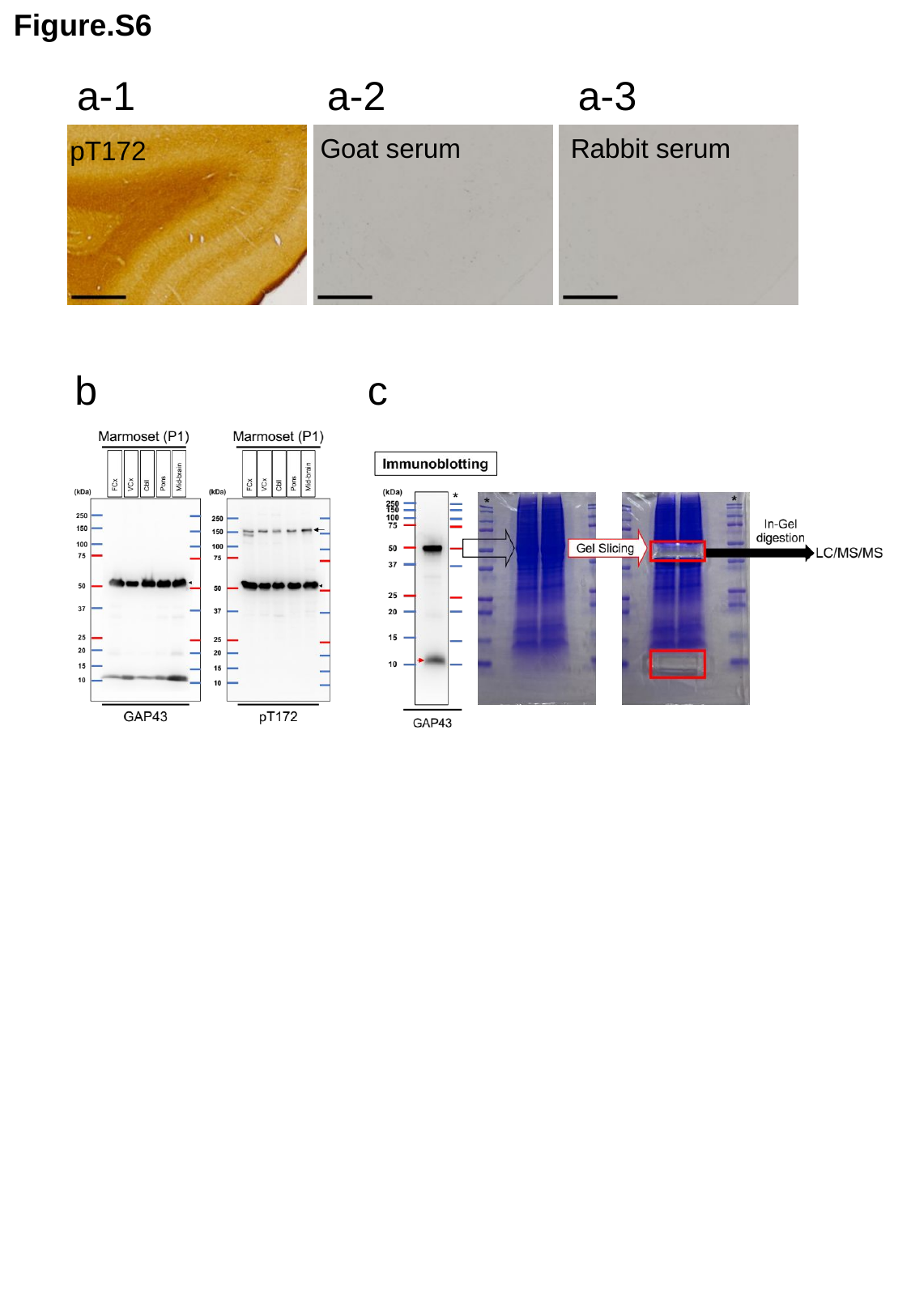

Figure.S6
a-1
a-2
a-3
Goat serum
Rabbit serum
pT172
b
c

Supplement: Supplementary file 7 — Additional file 7: Figure S6. Phospho-specificity of GAP-43 pT181 in the P1 marmoset. (a) Negative control immunostaining of P1 Marmoset visual cortex. pT172 pAb (a-1), secondary Ab alone (a-2; goat anti-rabbit Ab), and nonspecific IgG (a-3; rabbit serum). See also Fig. S3. Scale bar; 500 µm. (b) Immunoblot reactivity of pT172 Ab on a full-sized blot membrane. These results show the specificity of pT172Ab. (c) Phosphoproteomics procedure for the mid-brain including pons of P1 marmoset. The band corresponding to GAP-43 was cut out after SDS-PAGE of the sample, trypsinized in-gel, and subjected to MS analysis. *, molecular mass marker. Note that the GAP-43 immunoreactivity band (arrow in red) did not consist of the GAP-43 amino acid sequence by LC/MS/MS analysis, suggesting that it may be a non-specific reaction. The analyzed results are shown in Fig. 5p. [file 13041_2021_755_MOESM7_ESM.pptx]

Figure.S7

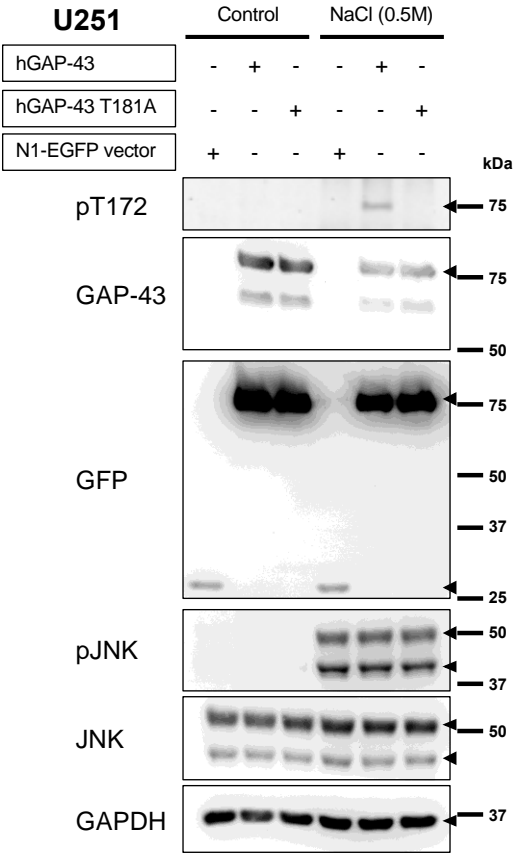

Supplement: Supplementary file 8 — Additional file 8: Figure S7. Stress-dependent JNK activation phosphorylates human GAP-43 T181 and is recognized by pT172Ab. Extracts from the transfected U251G cells were prepared at 30 min after osmotic stress (0.5 M NaCl) for JNK activation and immunoblotted using pT172, GAP-43, GFP, pJNK, JNK, and GAPDH Abs by the methods of Kawasaki et al. (2018). [file 13041_2021_755_MOESM8_ESM.pdf]
