# Supplementary material for: Phosphorylation of GAP-43 T172 is a molecular marker of growing axons in a wide range of mammals including primates
Source: Mol Brain. 2021 Apr 8;14:66. doi: 10.1186/s13041-021-00755-0 (PMC8034164; doi:10.1186/s13041-021-00755-0)
Supplement: Supplementary file 4 — Additional file 4: Figure S3.. Expression pattern of pT172 in the developing mouse brain. (a) Negative control immunostaining of E15 thalamocortical pathway. pT172 pAb (a-1), secondary Ab alone (a-2; goat anti-rabbit Ab), and nonspecific IgG (a-3; rabbit serum). Each concentration of proteins was 0.26 µg/ml. Scale bar: 200 µm. (b) Microscopic images of sagittal sections derived from various brain regions were DAB-stained using pT172. Boxes in (b) represent the regions enlarged in (c-g). pT172 succeeded in labeling nerve fibers under a growing state. Scale bar: 500 µm in (b), 200 µm in (c; in c-g). (h, i) Z-stack maximum-intensity projection images of basal nuclei (h) and OB (i). pT172 was more closely colocalized in the developing axons labeled by L1 than pan-GAP-43 in OC, LOT, and STR. GAP-43 and pT172 were detected in neurons of MCL and ONL (i). CP: cortical plate; LOT: longitudinal olfactory tract; MCL: mitral cell layer; OB: olfactory bulb; OCh: optic chiasm; ONL: olfactory nerve layer; Teg: longitudinal tegmental tracts; STR: striatum; PON: pons; EGL: external granular layer, PK: Purkinje cell layer. [file 13041_2021_755_MOESM4_ESM.pptx]

## Slide 1
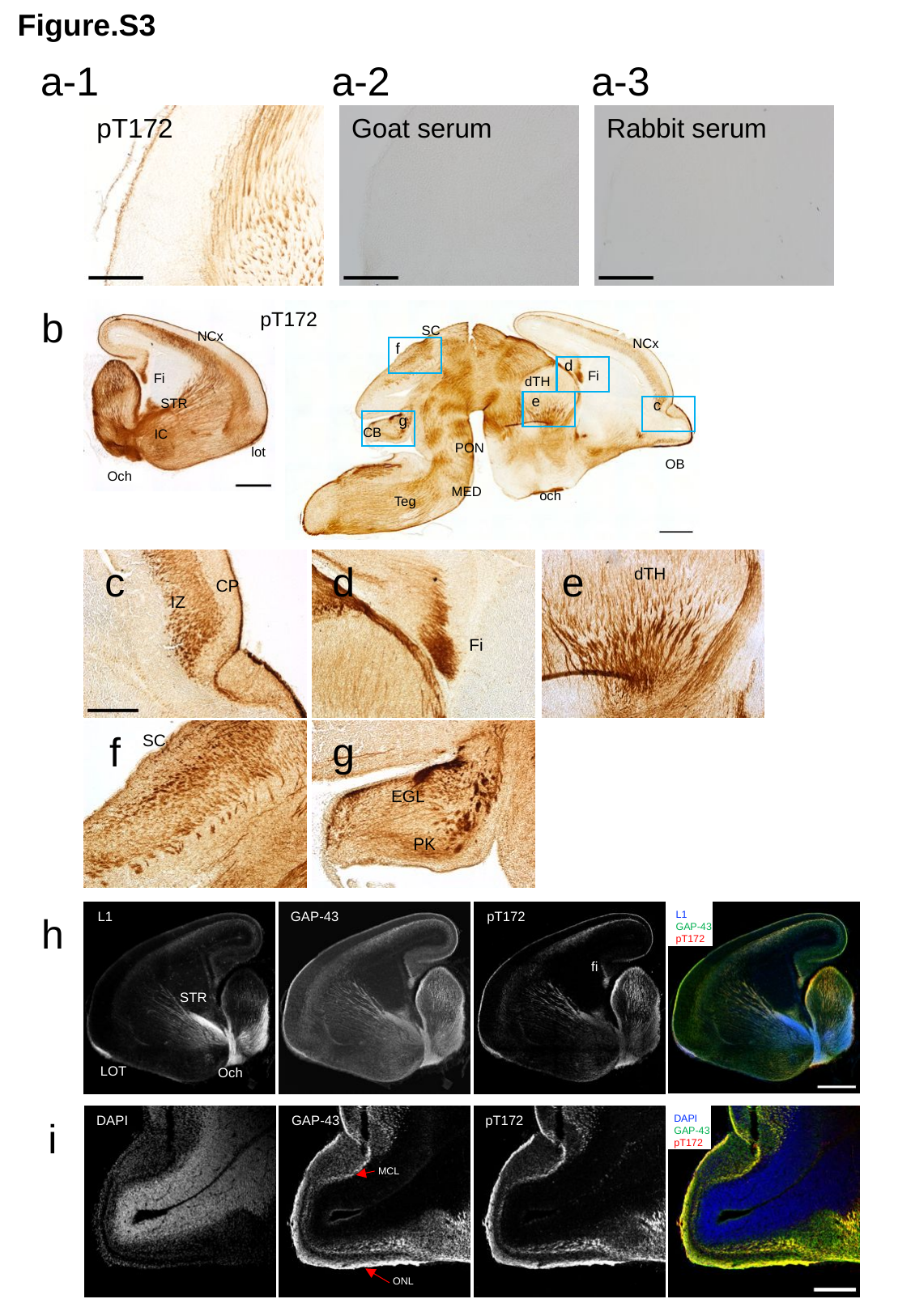

Figure.S3
a-1
a-2
a-3
pT172
Goat serum
Rabbit serum
b
pT172
SC
NCx
NCx
f
d
Fi
Fi
dTH
e
STR
c
g
CB
IC
PON
lot
OB
Och
MED
och
Teg
c
d
e
dTH
CP
IZ
Fi
f
g
SC
EGL
PK
h
L1
GAP-43
pT172
L1
GAP-43
pT172
fi
STR
LOT
Och
DAPI
GAP-43
pT172
DAPI
GAP-43
pT172
i
MCL
ONL
