# Supplementary material for: Phosphorylation of GAP-43 T172 is a molecular marker of growing axons in a wide range of mammals including primates
Source: Mol Brain. 2021 Apr 8;14:66. doi: 10.1186/s13041-021-00755-0 (PMC8034164; doi:10.1186/s13041-021-00755-0)
Supplement: Supplementary file 6 — Additional file 6: Figure S5. Amino acid sequences of GAP-43 surrounding the residue corresponding to S96 of rodents, in various mammalian species. S96 position of rodent GAP-43 is shown in red. See Discussion. [file 13041_2021_755_MOESM6_ESM.pdf]

Figure S5

```
Homo sapiens             89 EAAPATGSKPDEPGKAG 105
Macaca fascicularis      89 EAAPATGSKPDEPGKAG 105
Callithrix jacchus       89 EAAPATGTKPDETGKAG 105
Mustela putorius furo    90 DAIPASGPKPEESGKAG 106
Rattus norvegicus        90 DAAPATSPKAEEPSKAG 106
Mus musculus             90 DAAPATSPKAEEPSKAG 106
                                  *
```
